# Posttraumatic Symptoms in 3–7 Year Old Trauma-Exposed Children: Links to Impairment, Other Mental Health Symptoms, Caregiver PTSD, and Caregiver Stress

**DOI:** 10.1007/s10578-020-01093-3

**Published:** 2020-11-27

**Authors:** Matti Cervin, Alison Salloum, Leigh J. Ruth, Eric A. Storch

**Affiliations:** 1 Department of Clinical Sciences Lund, Child and Adolescent Psychiatry, Faculty of Medicine, Lund University, Sofiavägen 2D, 22241 Lund, Sweden; 2 University of South Florida, Tampa, FL USA; 3 Baylor College of Medicine, Houston, TX USA

**Keywords:** PTSD, Children, Parent, Caregiver, Network analysis, Stress

## Abstract

Few studies have examined how PTSD symptoms in young children are associated with other mental health symptoms and mood and functioning in caregivers. This is an important gap in the literature as such knowledge may be important for assessment and treatment. This study used network analysis to identify how the major symptom domains of PTSD in young trauma-exposed children were related to impairment, internalizing and externalizing symptoms, caregiver PTSD, and caregiver stress. Caregivers of 75 trauma-exposed 3–7 year old children reported on their child’s symptoms and impairment and their own PTSD symptoms and caregiver stress. A strong association between the child PTSD domains of intrusions and avoidance emerged, which is in line with theoretical notions of how PTSD onsets and is maintained in adolescents and adults. Externalizing child symptoms were strongly linked to PTSD-related impairment and caregiver stress, highlighting the need to carefully assess and address such symptoms when working with young trauma-exposed children. Internalizing symptoms were uniquely associated with all three of the major childhood PTSD symptom domains with further implications for assessment and treatment.

## Introduction

More than half of all children experience a traumatic event before adulthood (e.g., abuse, disasters, accidents) [1, 2] and 10–30% go on to develop posttraumatic stress disorder (PTSD) after such an event [3]. PTSD symptoms in children severely interfere with social and educational functioning [4–6] and drives a wide range of poor long-term outcomes, such as increased risk of depression, anxiety, somatic problems, and suicide [7, 8].

It is important to take into consideration caregiver and family factors when appreciating the development and maintenance of posttraumatic symptoms and PTSD in young children. Children are often dependent upon their caregivers to acknowledge when they experience distress and caregivers are key to seeking treatment. Furthermore, caregiver reactions to traumatic events predict PTSD development in children [9] and the persistence of symptoms [10]. Mental health symptoms among caregivers (e.g., PTSD, depression) have also been shown to mediate outcome for trauma-exposed children [11, 12]. A better understanding of how PTSD symptoms in young children are associated with caregiver PTSD and stress could inform assessment and treatment.

In the fifth edition of the Diagnostic and Statistical Manual of Mental Disorders (DSM-5), it was acknowledged that PTSD in young children may be expressed in ways that differ from adolescents and adults. For this reason, DSM-5 includes a preschool subtype of PTSD that shifts the focus to identifying more behaviorally expressed PTSD symptoms in children less than 7 years old. Another novelty in DSM-5, which applies for PTSD across the age range, is that a new cluster of PTSD symptoms was introduced alongside intrusions, hypervigilance/arousal, and avoidance. This cluster is called negative alterations in cognition and mood (NACM) and captures broader internalizing symptoms. For young children, one or more criteria of avoidance *or* NACM symptoms must be present; whereas for those above 6 years old, symptoms of *both* avoidance and NACM must be present. The new developmental subtype for young children may help clinicians and parents better identify the behaviorally expressed symptoms. However, challenges with assessing PTSD among young children remain as diagnostic interviews and self-report measures are typically only conducted with the caregiver. While caregivers’ observations are critical to understanding the young child’s symptomatology, caregivers may also be prone to over or under-reporting [13].

From a systemic perspective, PTSD symptoms in young children are embedded in the child’s environment. Caregiver PTSD and caregiver stress may be particularly important in this respect as caregiver mood and functioning may be connected to child symptoms in a variety of causal and mutually maintaining ways [14]. Network analysis is a theoretical and statistical framework developed to help outline complex associations among different aspects of functioning and behavior. Within network analysis, it is posited that different facets of human functioning and behavior are involved in a dynamical interplay through which one area of functioning directly affects other areas [15]. Network analysis in relation to childhood PTSD has been used only in relation to distinct (i.e., not on a cluster level) DSM-defined PTSD symptoms. Bartels et al. [16] analyzed self- and parent-report data from over 400 traumatized children and adolescents (aged 7–17 years) and estimated the network structure of DSM-5 PTSD symptoms. Symptoms in the NACM cluster introduced in DSM-5 emerged as most central (i.e., most strongly connected to other symptoms). Cao et al. [17] analyzed self-report, DSM-IV symptom data from a large sample of trauma-exposed adolescents (aged 12–16 years) and found that re-experiencing symptoms were central. Russell et al. [18] analyzed self-reported symptom data from a large group of children (aged 8–18 years) and found that avoidance and detachment symptoms emerged as partly distinct symptom clusters while symptoms of avoidance and intrusions were closely linked. De Haan et al. [19] used an international dataset with 2313 children (aged 6–18 years) and found that re-experiencing symptoms were central to the overall network of PTSD symptoms and that neither PTSD or depression symptoms were more strongly connected to dysfunctional posttraumatic cognitions.

Although informative, previous network studies have relied almost exclusively on PTSD symptom data, which precludes a broader examination of the ways in which PTSD in children are associated with broader mental health symptoms and other potentially important factors such as caregiver PTSD and caregiver stress. Furthermore, network analysis has not been used to examine posttraumatic symptoms in exclusively young children (i.e., ≤ 7 years). This is a notable gap as there is evidence to suggest that PTSD in this age group may be expressed differently and may be more strongly linked to factors in the environment such as caregiver mood and functioning. The purpose of this study was to identify how the major symptom domains of PTSD in young trauma-exposed children (aged 3–7 years) were associated with (1) each other, (2) broad internalizing and externalizing symptoms, (3) caregiver PTSD and caregiver stress, and (4) PTSD-related child impairment. All of these factors were modeled as a network of interconnected nodes and unique associations among nodes were outlined.

## Methods

### Participants, Procedure, and Ethics

A convenience sample of 79 caregivers seeking treatment for their young trauma-exposed child were recruited from a community-based mental health agency in the southeast of the United States for trauma-focused clinical trials [20, 21]. Four caregivers were excluded because of extensive missing data on study measures. No sibling pairs were included. Of the caregivers, 66 were mothers (88%), 3 were fathers, (4%) 4 were grandmothers (5%), 1 was an aunt (88%), and 1 a great aunt (1%). Included children were aged 3–7 years and had a mean age of 5.05 years (*SD* = 1.36; range: 3–7 years; 57% boys). The most common types of trauma were sexual abuse (33%) and domestic violence (32%) followed by physical abuse (8%), death (7%), illness/medical condition (5%), and accidents (4%). Fifty-six (75%) children had experienced interpersonal violence and 55 (73%) had experienced more than one trauma. The mean age of caregivers was 31.57 years (*SD* = 5.82) and 40 (53%) were employed. With respect to race, 53 children were White (71%), 17 children were African American (23%), 4 were Mixed Race (5%), and 1 was American Indian or Alaskan Native (1%). With respect to ethnicity, all participants were asked whether they identified as Hispanic or Latino, with 31 participants (41%) affirming this. Few caregivers reported not having experienced a trauma (*n* = 3; 4%). Fourteen caregivers reported one trauma (19%), 19 two traumas (25%), and 39 had experienced three or more traumas (52%). The most common types of traumas in the caregiver group were domestic violence (*n* = 18; 24%), sexual abuse (*n* = 11; 15%), death (*n* = 11; 15%), and physical abuse (*n* = 5; 7%). Inclusion and exclusion criteria for the present study have been reported in previous publications [20, 21].

The University of South Florida Institutional Review Board approved the study. The clinical trial was registered at clinicaltrials.gov (NCT01603563). Caregivers were provided written informed consent, children aged 7 were provided written assent, and younger children were provided a brief explanation of the study as written assent was waived for children aged 3–6 years. Given the challenges of assessing PTSD with young children (Leigh et al. 2016), caregivers were asked to complete assessment measures regarding their child’s posttraumatic stress symptoms and related problems, and they were also asked to complete measures of their own PTSD symptoms and caregiver stress/caregiver-child functioning.

### Measures

#### Trauma Symptom Checklist for Young Children (TSCYC)

TSCYC is a 90-item caregiver-report questionnaire assessing trauma and related symptoms in children from ages 3 to 12 [22]. Each item is rated on a scale from 1 (*not at all*) to 4 (*very often*) with higher ratings indicating more frequent symptoms. The scale yields three subscales directly related to PTSD (Intrusions, Avoidance, Arousal) and five scales entailing symptoms that frequently co-occur with childhood PTSD (Sexual Concerns, Dissociation, Anxiety, Depression, Anger/Aggression). In this study, the Intrusions, Avoidance, Arousal, and Dissociation scales were used. All scales exhibited adequate to excellent internal consistency (*a*s = 0.84, 0.86, 0.78, and 0.94, respectively).

#### Child Behavior Checklist (CBCL)

Broad internalizing and externalizing child mental health problems were assessed with the CBCL in which caregivers report on the presence of different child competencies and behavior problems each rated on a 0 (*not true*) to 2 (*very/often true*) scale with higher ratings indicating more severe problems. We used the pre-school version for the 3–5 year olds (100-items [23]) and the school-aged version for the 6–7 year olds (113-items [24]). The ratings are summed and transposed to t-scores (*M* = 50; *SD* = 10 in the general population) which yields eight symptom dimension scales and two broader scales reflecting overall internalizing (e.g., anxiety, depression) and externalizing (e.g., aggression, attention problems) symptoms. In this study, only these broader internalizing and externalizing scales were used.

#### Post-traumatic Checklist Scale (PCLS)

PCLS was used to measure caregiver PTSD. It is a 17-item self-report scale with sound psychometric properties covering PTSD symptoms in adults with each item being rated on a scale from 1 (*not at all*) to 5 (*extremely*) [25]. The scale has sound psychometric properties (Weathers et al., 1993) and generates a total score and three subscales corresponding to the symptom clusters described in DSM-IV. The scores of re-experiencing (*a* = 0.91), avoidance (*a* = 0.84), and arousal (*a* = 0.85) scales all showed good internal consistency in this sample, as did the total score (*a* = 0.95).

#### Parenting Stress Index-Short Form (PSI-SF)

The PSI-SF was used as a measure of caregiver-child functioning. It is a caregiver-reported scale that includes 36 items rated on a 1 (*strongly disagree*) to 5 (*strongly agree*) scale with higher scores indicating higher levels of stress [26]. It yields three subscales: (1) Parental Distress (the caregiver’s experience of his/her child-rearing competencies); (2) Parent–Child Dysfunctional Interaction (the caregiver’s experience of interaction with the child as non-reinforcing and of the child failing to meet caregiver expectations); (3) Difficult Child (the caregiver’s experience of the child’s temperament and degree of non-compliance) as well as a total score with higher scores indicating more caregiver stress. Only the total score was used in this study and it showed excellent internal consistency (*a* = 0.93).

#### Diagnostic Infant Preschool Assessment (DIPA)

DIPA is a semi-structured caregiver interview assessing psychiatric symptoms in young children (1–6 years) according to DSM-IV criteria [27]. In the present study, the impairment section of the PTSD module of the instrument was used to assess the degree of impairment caused by PTSD in relation to caregivers, siblings, peers, daycare provider/teacher, everyday behavior, and child’s distress related to impairment. The six impairment items were endorsed as yes (= 1) or no (= 0) to create a total impairment score ranging from 0 to 6. Further, as part of a sensitivity analysis, the DIPA scales for re-experiencing, avoidance, and arousal were used.

### Statistical Analysis

All variables were examined for normality and skewness. Several variables were non-normal and the impairment variable was ordinal in nature. Thus, we computed partial correlations using polychoric correlations which estimates correlations among theoretically normally distributed variables using ordinal data. Second, because network analysis is sensitive to problems with multicollinearity (i.e., highly correlated variables), we inspected the zero-order polychoric correlations for correlations > 0.80. All of the adult PTSD subscale variables were correlated > 0.80 and we decided to group these into a single variable representing caregiver PTSD. Child re-experiencing and child avoidance were also highly correlated (0.76) but we decided to keep these variables as separate variables in the network.

The network structure of the study variables was estimated using the R-package *bootnet*. The proportion of missing data was low (1.6%) and missing data were handled within the model using pairwise deletion. Polychoric correlations were estimated based on the reasoning outlined above. The networks were regularized (i.e., edges were shrunk so that small edges were set to zero) using graphical LASSO [28]. Within a network framework, each unique variable-to-variable association is referred to as an edge and these are depicted as lines that connect variables (with variables being called nodes). All edges in this study correspond to regularized partial correlation coefficients which can take on a value between –1 and 1. All networks were plotted using the Fruchterman-Reingold algorithm so that strongly connected nodes were placed closely and nodes with many and strong connections to other nodes centrally while also minimizing overlap of nodes and edges.

Because each edge is a point estimate based on the sample and without an estimated uncertainty interval that reflects where the true effect in the target population may lie, the accuracy of edges were examined by running 1000 bootstraps which were used to produce a 95% confidence interval for each edge. Non-parametric bootstraps were used. The bootstrapped-based estimates were then employed to estimate whether there were statistically significant differences between edge pairs.

We were interested in which variables that connected child and caregivers variables and used the R-package *networktools* to estimate bridge expected influence which is an estimate of the degree of positive associations that a node within a predefined cluster of nodes has to nodes in other clusters [29]. Thus, bridge expected influence is a measure of the degree to which a node links two node clusters. To estimate bridge expected influence for each node, we used predefined clusters of nodes for child (PTSD domains, dissociation, CBCL internalizing and externalizing, and impairment) and caregiver variables (PTSD and caregiver stress), respectively. We used 1-step estimation that only takes into account direct links between the target node and nodes outside of the target node’s predefined node cluster.

We were also interested in which nodes were most closely linked to PTSD-related child impairment. This was analyzed by simply examining the edges between this node and other nodes in the network.

Last, because of the pure reliance on caregiver-reported child symptoms, we carried out two sensitivity analyses. First, we examined whether the association between child and caregiver variables varied based on the degree to which the caregiver experienced PTSD symptoms. For this analysis, the sample was split according to the median value (= 33) on the PCLS scale which resulted in 32 caregivers being classified as having low PTSD symptoms and 33 caregivers as having high PTSD symptoms. Correlations between the child symptom scales and caregiver stress were then computed in each group separately; correlations between child symptom scales and PCLS were not performed as the sample was split on PCLS. Second, we estimated a network in which the clinician-rated DIPA scores, instead of the caregiver-reported TSCYC scores, were used as indicators of the child PTSD domains. The dissociation variable was omitted as this domain is not assessed in DIPA. Networks based on TSCYC and DIPA, respectively, were estimated and descriptively compared because of the low statistical power.

## Results

**Fig. 1:**
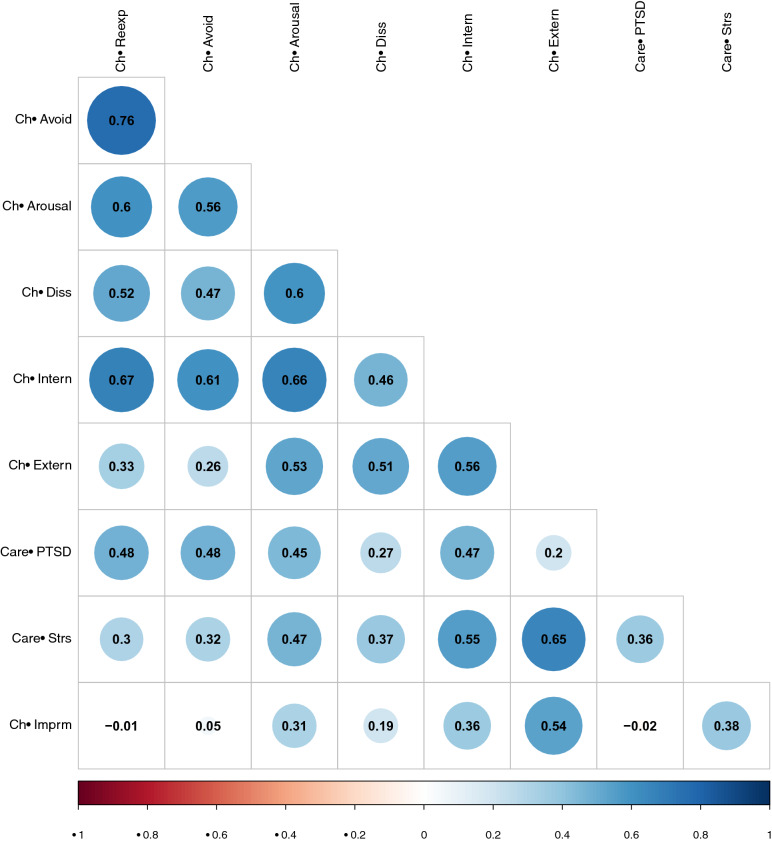
Zero-order polychoric correlations among study variables. Correlations marked with a circle are statistically significant at a *p* < .01 level

Means and standard deviations for all measures are presented in Table 1. For comparison purposes, normative scores on TSCYC, CBCL, PCLS, and PSI-SF from the general population are also presented [22, 30, 31]. For TSCYC, the mean scores for the children in the present study were more than one standard deviation above the normative scores for all scales except for dissociation. Similar results were found on the CBCL broadband scales. Caregivers in the present study had elevated scores on the PSI-SF and PCLS but not as elevated as the TSCYC/CBCL scores of their children. The zero-order polychoric correlations for all study variables are presented in Fig. 1.

**Table 1:** Means and standard deviations for the measures included and normative data from the general population

	*n*	*M* (*SD*)	Normative data *M* (*SD*)
Child measures			
TSCYC-Re-experiencing	75	16.6 (5.1)	10.0 (1.5)/10.5 (2.1)^a^
TSCYC-avoidance	75	17.2 (6.1)	9.8 (1.7)/10.2 (2.1)^a^
TSCYC-arousal	75	20.7 (5.7)	11.9 (3.1)/12.5 (3.4)^a^
TSCYC-dissociation	75	15.7 (6.7)	10.9 (2.8)/11.6 (3.3)^a^
CBCL-internalizing	75	64.2 (9.9)	50 (10)
Caregiver measures			
PCLS-total Score	65	36.4 (16.6)	29.4 (12.9)^b^
PSI-SF	75	83.96 (23.3)	73.4 (25.3)^c^
Clinician-rated measures			
DIPA impairment	74	4.1 (1.5)	
DIPA Re-experiencing	74	3.3 (1.2)	
DIPA avoidance	74	2.7 (1.7)	
DIPA arousal	74	3.5 (1.0)	

*TSCYC* trauma symptom checklist for young children, *CBCL* Child Behavior Checklist, *DIPA* diagnostic infant preschool assessment, *PCLS* Post-Traumatic Checklist Scale, *PSI-SF* Parenting Stress Index-Short Form

^a^For 3–4 and 5–9 year olds, respectively; Briere (2005)

^b^Blanchard et al. (1996)

^c^Reitman et al. (2002)

### Association Network of Study Variables

**Fig. 2:**
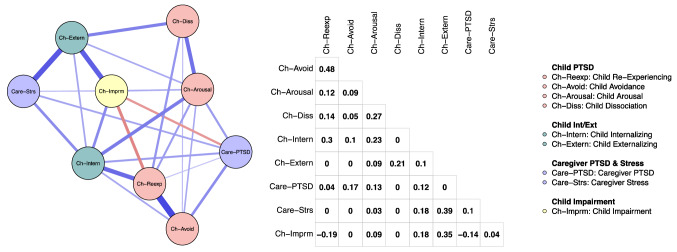
The network structure of the study variables and the partial correlation matrix. Each variable is depicted as a node (a circle). Lines (edges) between nodes are unique associations (partial *r*s). Blue edges depict a positive association. Red edges depict a negative association. Wider and more saturated edges depict a stronger association. The coloring of nodes is based on pre-defined groups of nodes (Color figure online)

**Fig. 3:**
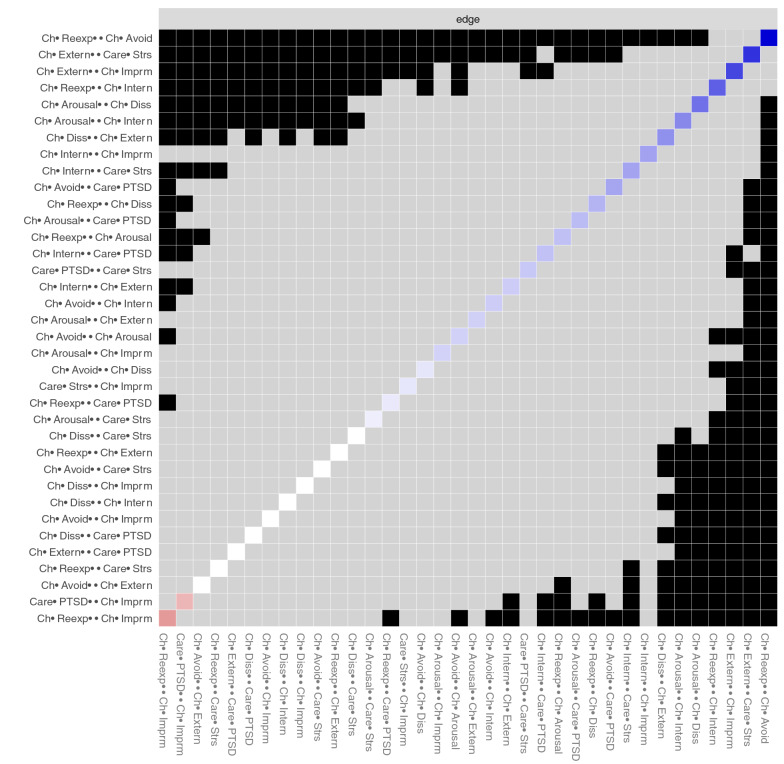
Statistically significant differences between edge weights. Black boxes indicate that there is a statistically significant different between the strength of two edges. Edges are listed according to their strength with the strongest each at the top of the y-axis

The network structure of the study variables is depicted in Fig. 2. Statistically significant differences in edge weights are presented in Fig. 3. Two edges were significantly stronger than a majority of all other possible edges (the variable set contained 36 possible edges): child re-experiencing—child avoidance (edge weight [EW] = 0.48) and child externalizing—caregiver stress (EW = 0.39). Further child externalizing—child impairment (EW = 0.35) and child re-experiencing—child internalizing (EW = 0.30) were stronger than 19 and 15 of the other possible edges, respectively. Two negative edges emerged: caregiver PTSD—child impairment (EW =  − 0.14) and child re-experiencing—child impairment (EW =  − 0.19). These edges had a significantly lower value than 12 and 17 of the other possible 35 edges, respectively.

### Nodes Bridging Caregiver and Child Nodes

To test which nodes were most important for connecting the child and caregiver nodes, we estimated bridge expected influence for each of the nodes. Among the child nodes, internalizing (bridge expected influence estimate = 0.29) and externalizing (bridge expected influence estimate = 0.39) stood out as the most important bridge nodes to the caregiver nodes of PTSD and stress. Avoidance (bridge expected influence estimate = 0.17) and arousal (bridge expected influence estimate = 0.16) were also acting as bridge nodes. PTSD-related child impairment had a negative bridge expected influence estimate (− 0.09) with a positive association with caregiver stress (EW = 0.04) and a negative association with caregiver PTSD (EW =  − 0.14). In respect of caregiver nodes, parenting stress (bridge expected influence estimate = 0.64) acted as a link between child and caregiver nodes. Caregiver PTSD (bridge expected influence estimate = 0.32) did also act as a link but had both positive and negative associations with child nodes.

### Nodes Linked to Child Impairment

The partial correlation matrix and the network structure revealed that child externalizing (EW = 0.35), child internalizing (EW = 0.18), child arousal (EW = 0.09), and caregiver stress (EW = 0.04) were significantly and positively associated with child impairment. Child re-experiencing (EW =  − 0.19) and caregiver PTSD (EW =  − 0.14) were significantly negatively associated with child impairment.

### Sensitivity Analyses

In Table 2 are correlations between child PTSD domains and caregiver stress among caregivers with high versus low PTSD scores. There were no statistically significant differences for correlation coefficients in the two groups but statistical power was low. The largest point estimate differences emerged for the correlation between caregiver stress and arousal (difference: 0.28) and caregiver stress and dissociation (difference: 0.22).

**Table 2:** Spearman rank-order correlations between caregiver stress and child factors in caregivers with low (< 33 points) respectively high values (≥ 33 points) on the caregiver PTSD scale (PCLS)

	Low caregiver PTSD *n* = 32	High caregiver PTSD *n* = 33	Statistically significant difference (alpha = 0.05)
Re-experiencing	0.14	0.22	No
Avoidance	0.37*	0.10	No
Arousal	0.37*	0.65**	No
Dissociation	0.39*	0.17	No
Internalizing symptoms	0.47**	0.50**	No
Externalizing symptoms	0.75**	0.62**	No

*PCLS* Post-Traumatic Checklist Scale

*Indicates *p* < 0.05

**Indicates *p* < 0.01

**Fig. 4:**
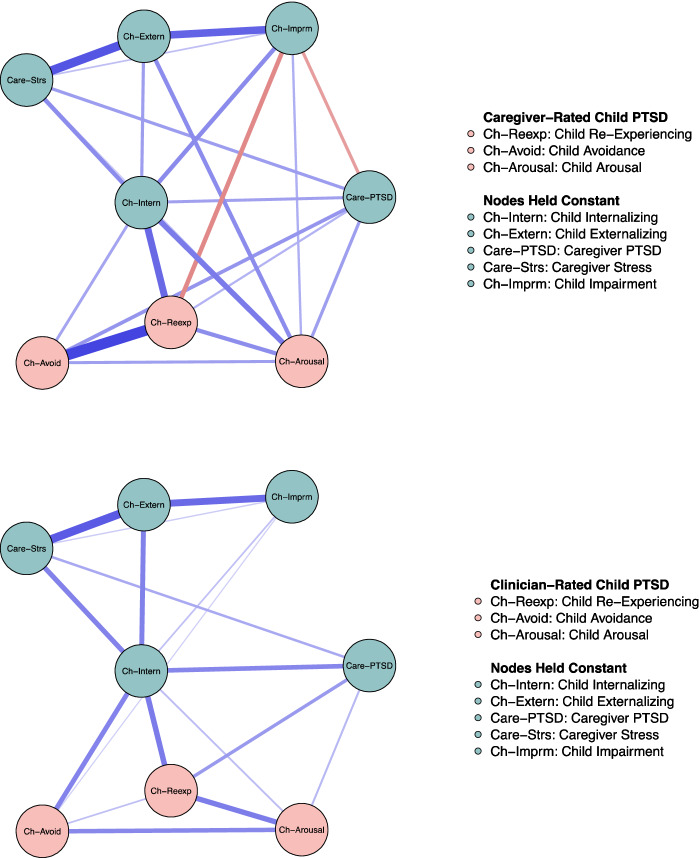
Networks estimated using caregiver-reported (top) and clinician-rated (bottom) child PTSD domains. Each variable is depicted as a node (a circle). Lines (edges) between nodes are unique associations (partial *r*s). Blue edges depict a positive association. Red edges depict a negative association. Wider and more saturated edges depict a stronger association. The coloring of nodes is based on pre-defined groups of nodes (Color figure online)

The networks estimated using DIPA (clinician-rated) and TSCYC (caregiver-report) for the child PTSD domains are presented in Fig. 4. A descriptive comparison revealed that associations among the PTSD domains and between PTSD domains and other nodes were attenuated when clinician-rated data were used. However, in both networks, all PTSD domains were positively linked to each other and to internalized child symptoms. Further, in both networks, caregiver PTSD was linked to child re-experiencing and child arousal. The strong edge between child re-experiencing and child avoidance in the caregiver-rated network appeared to be clearly attenuated in the clinician-rated network.

## Discussion

This study examined associations among the major symptom domains of childhood PTSD in trauma-exposed children younger than 7 years old and how these domains were associated with broader internalizing and externalizing child symptoms, dissociation, caregiver PTSD, caregiver stress, and PTSD-related child impairment. Network analysis was chosen to capture the complexity assumed to underlie these associations. The sample size was modest and results are in need of replication. Nevertheless, some of the results appear robust. The major findings were: (1) a strong association between child re-experiencing and child avoidance as reported by the caregiver; (2) that broad externalizing child symptoms were more strongly associated with caregiver stress and PTSD-related child impairment than PTSD symptoms; (3) a clear association between child re-experiencing (and to some degree child arousal) and broad internalizing child symptoms.

The close connection between child re-experiencing and avoidance aligns well with theoretical notions of PTSD. Intrusive and avoidance symptoms, when associated with a traumatic event, are unique only to PTSD and acute stress disorder. Ehlers and Clark’s [32] cognitive model of PTSD helps explain this relationship such that, as intrusion symptoms occur, avoidance coping occurs which in turn prevent opportunities to process the traumatic memories and appraise the current perceived threats; thus, PTSD is maintained. While young children may not engage in the same cognitive appraisals as older children, children younger than 7 years old can engage in active efforts to avoid intrusive symptoms associated with traumatic events. Findings from this study, highlighting a clear association between intrusions and avoidance, are also consistent with Russell et al. [18] who found using network analysis that intrusion and avoidance symptoms were closely linked among different age groups (children 8 to 13 and youth 14 to 18 years). Thus, a connection between intrusions and avoidance has now been (preliminary) established across childhood and adolescence. However, it is important to note that this connection was much less pronounced when we estimated a network using clinician-rated child PTSD domains. Future work should consider which are the best ways to assess re-experiencing and avoidance symptoms in young children, particularly for children in early stages of verbal and emotional development.

Aggressive and externalizing behaviors in children exposed to a trauma are well documented [33, 34]. Our findings suggest that such symptoms may play a prominent role in symptom expression and functioning and that these symptoms may be more important for impairment and caregiver stress than symptoms included in the diagnostic description of PTSD. The CBCL broadband scale of externalizing symptoms include symptoms of rule-breaking behavior and aggression. Scheeringa et al. [35] found that oppositional defiant disorder (ODD: an externalizing problem) was the most common comorbid disorder with PTSD among preschoolers exposed to trauma. When comparing preschoolers with repeated trauma, single incident trauma, and hurricane related trauma, Scheeringa [36] found that ODD, prior to trauma exposure, was more prevalent than other psychiatric conditions in the repeated trauma group. This is in line with evidence showing that externalizing symptoms in children, present before trauma-exposure, are a risk factor for adult PTSD (Koenen et al. 2007). Seventy-three percent of the children in our sample experienced more than one trauma which may contribute to the strong associations between externalizing problems, child impairment, and caregiver stress; although, it is unknown if there were other child, family, and/or environmental factors prior to the trauma that also contributed to these associations as well as to the repeated traumas.

Although the new DSM-5 PTSD symptom cluster of NACM was not directly assessed in the current study, the broad assessment of internalizing symptoms using the CBCL overlap with the observable NACM symptoms in young children (e.g., increase in negative emotional states, persistent decrease in positive emotions, diminished interests in activities, including play, and social withdrawal). In this study, internalizing symptoms had unique associations with all of the three major symptom domains of childhood PTSD, with a particularly strong association with re-experiencing. These findings lend partial support to the inclusion of NACM in the description of PTSD in young children, even though the NACM PTSD symptom cluster for young children does not include specific cognitive symptoms that are included for older children and adults (e.g., not able to remember aspect of the trauma, negative beliefs and distorted cognitions).

Dissociation was included because its role in childhood PTSD is not well understood. The present results suggest that there may be a unique link between dissociation and externalizing symptoms. It is possible that this association reflects the severe dysregulation that can occur with dissociation [37] and the difficulties dysregulation can pose in the caregiver -child relationship. Dissociation was also linked to arousal, re-experiencing and avoidance, although the association with arousal was most pronounced. Taken together, findings provide preliminary support for the subtype of dissociative symptoms in DSM-5 rather than dissociation being a core feature of PTSD in young children, and that it may be equally (or more) associated with externalizing symptoms as with PTSD symptoms.

Consistent with prior research [38, 39], caregiver and child PTSD were associated, although the unique associations in this study were quite modest; however, the associations were present also in the network based on clinician-rated child PTSD domains. In network analysis, unique associations are estimated by controlling for all other associations in the network. This comprehensive control attenuated the associations between caregiver and child PTSD in the network based on caregiver-report (see Fig. 1 for zero-order correlations). This suggests that it is important to consider which factors other than PTSD that can affect the relationship between caregiver and child PTSD. However, despite a comprehensive control for other variables, caregiver PTSD was uniquely linked to child re-experiencing, avoidance, and arousal, and for re-experiencing and avoidance this finding replicated using clinician-rated child PTSD domains. It is likely that, given the high percentage of exposure to domestic violence for caregiver and children, shared trauma may be contributing to these connections. Indeed, caregiver-child dyads with histories of interpersonal trauma have shown higher overlap of PTSD symptoms than dyads with other types of trauma [38]. Caregivers struggling with exposure to domestic violence may also be challenged with having to adjust without the partner to new parenting strategies, especially for the child’s disruptive externalizing behaviors. Both maternal PTSD symptoms from interpersonal partner violence and restrictive and punitive parenting practice mediate caregiver exposure and young children’s internalizing and externalizing behaviors [40]. It is also plausible that the association between caregiver and child PTSD symptoms represents shared genetic vulnerability [41].

The negative associations between child re-experiencing and child impairment and caregiver PTSD and child impairment merit mention. There were no statistically significant zero-order correlations between these variables. Rather, the negative associations in the network based on caregiver-rated child PTSD domains were driven by that re-experiencing and caregiver PTSD are positively associated with other nodes in the network which in turn are positively associated with impairment (e.g., internalizing symptoms). Thus, if the node representing internalizing symptoms is held constant, the zero-order null association between, for example, re-experiencing and impairment, will turn negative. That is, when we account for internalizing symptoms, there is a negative association between re-experiencing and impairment. Again, because of the small sample, these associations should be interpreted carefully and no negative associations between these variables emerged in the network based on clinician-rated child PTSD domains. Nevertheless, the present results suggest that re-experiencing and caregiver PTSD, when controlling for broader internalizing child symptoms and other important factors, are not strongly positively linked to PTSD-related impairment in children younger than 7 years.

A major limitation of this study was the small sample that introduces uncertainties about the replicability of results. The data were collected as part of a treatment study and thus the sample was a convenience sample. We tried to account for the small sample by only interpreting the most robust findings and only specific edges and not overall measures such as strength centrality of each node. Another major limitation was the sole reliance on caregiver-report for child symptoms. Caregiver-child agreement of child PTSD symptoms correlates only in the moderate range [42, 43]. Thus, the caregiver-rated child PTSD symptoms may only partially capture PTSD symptoms as they are experienced by children in this young age group. It is possible or even probable that symptoms and prior experiences of the caregiver (e.g., own trauma) affects how behaviors and symptoms in their child are interpreted. How to best assess and measure PTSD symptoms and other internalized symptoms in young children is a challenge to the field and at present no perfect solutions are available. Third, because of the cross-sectional nature of the data, the reasons for the outlined associations remain elusive. Last, broadband CBCL scales were used and not the more specific subscales such as anxious/depressed and somatic complaints. Future research using larger samples should consider including more fine-grained scales of internalizing and externalizing symptoms.

## Summary

This study examined how the major symptom domains of childhood PTSD were associated, both with each other and with PTSD-related impairment, caregiver PTSD, and caregiver stress. We found strong associations between externalizing child symptoms and PTSD-related impairment and caregiver stress and it is advised that assessment/treatment of childhood PTSD includes assisting caregivers with strategies on how to help children exhibiting disruptive behaviors after traumatic events. We also found a strong association between intrusions and avoidance, which is in line with theoretical notions of how PTSD onsets and is maintained in adolescents and adults and suggests that similar mechanisms may be involved in young children. However, this was not replicated using clinician-rated child PTSD domains and should therefore be interpreted carefully. Broad internalizing symptoms were uniquely associated with all three of the major childhood PTSD symptom domains using both caregiver- and clinician-rated child PTSD symptoms. This implies that broader internalizing symptoms are closely linked to PTSD symptoms in young children, lending support that NACM in DSM-5 is also relevant in the youngest age range. Replication of results with larger samples and by type of trauma are needed for the generalizability of findings. Further, subgroup analyses across sexes and ages may yield more insight into developmental differences/similarities in how PTSD is expressed in young children. Future research is needed examining how PTSD symptoms in children and caregivers affect each other over time, including during treatment, and what role externalizing symptoms and caregiver stress play in the maintenance and amelioration of PTSD symptoms in young children.
